# Ethanol Extract of *Rosa laevigata Michx*. Fruit Inhibits Inflammatory Responses through NF-κB/MAPK Signaling Pathways via AMPK Activation in RAW 264.7 Macrophages

**DOI:** 10.3390/molecules28062813

**Published:** 2023-03-20

**Authors:** Hongtan Wu, Tingting Lin, Yupei Chen, Fangfang Chen, Shudi Zhang, Haiyue Pang, Lisen Huang, Chihli Yu, Gueyhorng Wang, Chun Wu

**Affiliations:** 1 Department of Public Health and Medical Technology, Xiamen Medical College, Xiamen 361023, China; 2 Engineering Research Center of Natural Cosmeceuticals, College of Fujian Province, Xiamen 361023, China; 3 Department of Pharmacy, Xiamen Medical College, Xiamen 361023, China; 4 Department of Clinical Medicine, Xiamen Medical College, Xiamen 361023, China

**Keywords:** EFR, LPS, anti-inflammatory, AMPK/MAPK/NF-κB cascade

## Abstract

The fruit of *Rosa laevigata Michx*. (FR), a traditional Chinese herb utilized for the treatment of a variety diseases, has notably diverse pharmacological activities including hepatoprotective, anti-oxidant, and anti-inflammatory effects. Despite ongoing research on illustrating the underlying anti-inflammatory mechanism of FR, the principal mechanism remained inadequately understood. In this study, we investigated in depth the molecular mechanism of the anti-inflammatory actions of the ethanol extract of FR (EFR) and its potential targets using lipopolysaccharide (LPS)-stimulated RAW 264.7 macrophages in vitro. We showed that EFR effectively ameliorated the overproduction of inflammatory mediators and cytokines, as well as the expression of related genes. It was further demonstrated that LPS-induced activation of nuclear factor kappa B (NF-κB) and mitogen-activated protein kinases (MAPKs) were significantly inhibited by pretreatment with EFR, accompanied by a concomitant decrease in the nuclear translocation of the p65 subunit of NF-κB and activator protein 1 (AP-1). In addition, EFR pretreatment potently prevented LPS-induced decreased phosphorylation of adenosine monophosphate-activated protein kinase (AMPK). Our data also revealed that the activation of AMPK and subsequent inhibition of the mammalian target of the rapamycin (mTOR) signaling pathway was probably responsible for the inhibitory effect of EFR on LPS-induced inflammatory responses, evidenced by reverse changes observed under the condition of AMPK inactivation following co-treatment with the AMPK-specific inhibitor Compound C. Finally, the main components with an anti-inflammatory effect in EFR were identified as madecassic acid, ellagic acid, quinic acid, and procyanidin C1 by LC–MS and testified based on the inhibition of NO production and inflammatory mediator expression. Taken together, our results indicated that EFR was able to ameliorate inflammatory responses via the suppression of MAPKs/NF-κB signaling pathways following AMPK activation, suggesting the therapeutic potential of EFR for inflammatory diseases.

## 1. Introduction

Inflammation is a complex pathophysiological response against tissue injury and infectious pathogens. Activated macrophages are known to play pivotal roles in mediating several immunopathological conditions by inducing the release of inflammatory mediators, such as nitric oxide (NO) and prostaglandin E_2_ (PGE_2_), and cytokines including tumor necrosis factor-α (TNF-α), interleukin (IL)-1β, and IL-6 [1]. However, hypernomic inflammation, mainly characterized by the overproduction of these immunosignaling molecules, has been proven to be deleterious and involved in the pathogenesis of various inflammatory diseases, for example arthritis, neurodegenerative diseases, and even cancer [2,3]. Herein, developing efficient drugs from natural derived extracts/compounds that could block the production of these inflammatory mediators and cytokines would be a promising therapeutic strategy for inflammatory diseases.

The LPS-stimulated macrophage model has been widely used for evaluating the anti-inflammatory activities of various synthetic or naturally derived substances in vitro [4,5]. LPS treatment causes the activation of several intracellular signaling pathways containing the I kappa B kinase (IKK)/NF-κB pathway and the MAPK pathway, and subsequent activation of a series of transcription factors containing NF-κB and AP-1, which mediate the expression of genes that encode inflammatory mediators and cytokines [6,7,8]. NF-κB plays a critical part in the regulation of immune responses. Under unstimulated conditions, NF-κB is retained in the cytoplasm in an inactive form by interaction with inhibitor κBα (IκBα) [9]. Upon stimulation with LPS, NF-κB is activated via the phosphorylation of IKKs, which in turn promotes the phosphorylation and ubiquitin-dependent degradation of IκBα, resulting in the translocation of NF-κB into the nucleus, where it binds to the κB sites in the promoter regions of multiple genes and enhances the production of inflammatory mediators and cytokines [10]. The activation of NF-κB is also regulated by the MAPKs, consisting of c-Jun N-terminal kinase (JNK), p38 MAPK, and extracellular-regulated protein kinase (ERK), which modulate another transcriptional regulator AP-1 by phosphorylation, ultimately leading to aggravated inflammatory reactions [11,12,13]. In addition, several intracellular kinases, such as AMPK, phosphatidylinositol 3-kinase (PI3K), and protein kinase B (AKT), have been implicated in the transcriptional regulation of inflammatory genes [14,15]. Hence, the suppression of inflammatory gene expression via the above and other cellular kinases or signaling pathways should be an efficacious strategy for the development of anti-inflammatory therapeutic drugs [14,16].

*Rosa laevigata Michx*., a famous medicinal plant belonging to the *Rosaceae* family, is mainly distributed throughout southern China. Its fruit, known as a commonly used traditional Chinese medicine, is widely used to treat chronic cough, frequent micturition, hyperpiesia, and dermatologic diseases [17,18]. Previous studies have demonstrated that the fruit has a variety of medicinal values and health benefits including anti-oxidant, hepatoprotective, and anti-inflammatory activities [19,20,21]. Nevertheless, among them, to the best of our knowledge, the principle mechanism of the anti-inflammatory properties remains unclear. Therefore, the aim of the present study is to further evaluate the anti-inflammatory effect of EFR and provide key insights into the potential mechanism underlying its actions.

## 2. Results

### 2.1. EFR Inhibits LPS-Induced Production of NO and PGE_2_ via the Suppression of iNOS and COX-2 Expression

**Figure 1:**
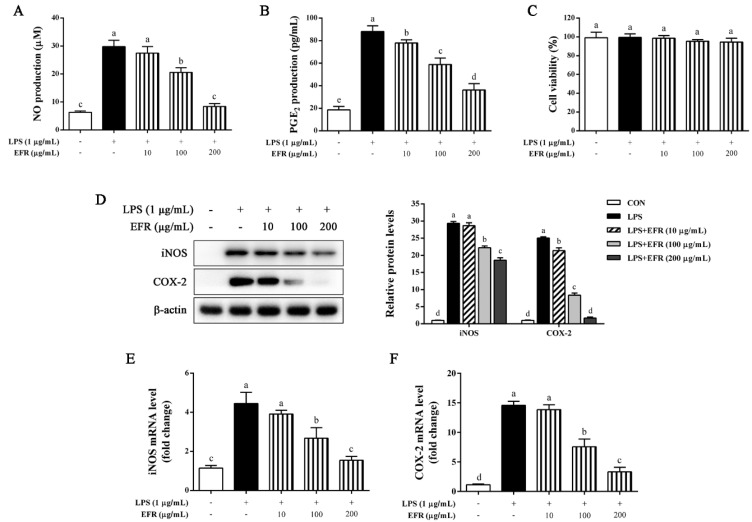
Effect of EFR on LPS-induced NO and PGE_2_ production and iNOS and COX-2 expression in RAW 264.7 macrophages. RAW 264.7 macrophages were pretreated with EFR (10, 100, and 200 μg/mL) for 2 h and then stimulated with or without LPS (1 μg/mL) for 24 h. (**A**) The nitrite concentration in the cultured media was determined as an indicator of NO production by Griess reaction. (**B**) PGE_2_ production in the cultured media was determined by ELISA. (**C**) The cell viability was determined by MTT assay. (**D**) The protein levels of iNOS and COX-2 were determined by Western blot analysis. β-actin was used as an endogenous control. Relative intensity of the immunoreactive bands was analyzed using the Image J software. The mRNA levels of iNOS (**E**) and COX-2 (**F**) were determined by qPCR. Results are shown as the mean ± SD of three independent experiments. The different letters represent the statistical differences at *p* < 0.05 among the groups by Duncan’s multiple range test.

The NO inhibitory effect specially serves as a measure for estimating the effectiveness of anti-inflammatory agents [22]. To investigate the anti-inflammatory effect of EFR, we determined the nitrite concentration in the cultured media in LPS-stimulated RAW 264.7 macrophages. As shown in Figure 1A, EFR markedly reduced NO production, which was measured as nitrite concentration and resulting from LPS treatment in a dose-dependent manner. In addition to NO, the level of PGE_2_ was also significantly decreased by EFR pretreatment in LPS-stimulated RAW 264.7 macrophages in a dose-dependent manner (Figure 1B). Further MTT assay indicated that EFR had unapparent cytotoxicity at concentrations ranging from 0 to 200 μg/mL (Figure 1C). iNOS and COX-2 are two vital enzymes for the synthesis of NO and PGE_2_, respectively [16]. As can be seen in Figure 1D–F, both protein and mRNA expressions of iNOS and COX-2 induced by LPS were prominently inhibited by EFR pretreatment in a dose-dependent manner. These results demonstrated that the inhibitory effect of EFR on the production of NO and PGE_2_ was probably attributed to transcriptional down-regulation of iNOS and COX-2 genes, not cytotoxic effect.

### 2.2. EFR Suppresses LPS-Induced Production of Inflammatory Cytokines

**Figure 2:**
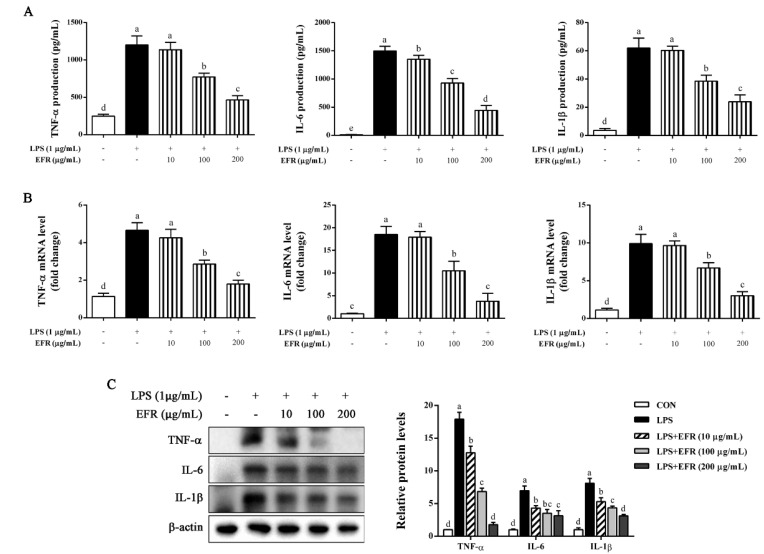
Effect of EFR on LPS-induced TNF-α, IL-6, and IL-1β production and expression in RAW 264.7 macrophages. RAW 264.7 macrophages were pretreated with EFR (10, 100, and 200 μg/mL) for 2 h and then stimulated with or without LPS (1 μg/mL) for 24 h. The secretion (**A**), mRNA (**B**), and intracellular protein (**C**) levels of TNF-α, IL-6, and IL-1β were determined by ELISA, qPCR, and Western blot analysis, respectively. IL-1β refers to full-length pro-IL-1β. Results are shown as the mean ± SD of three independent experiments. The different letters represent the statistical differences at *p* < 0.05 among the groups by Duncan’s multiple range test.

TNF-α, IL-6, and IL-1β are known to be early secreted and predominantly produced inflammatory cytokines involved in the pathogenesis of inflammation [23]. Thus, we determined the effect of EFR on the secretion of these inflammatory cytokines in LPS-stimulated RAW 264.7 macrophages. Our ELISA results illustrated that EFR pretreatment noteworthily and dose-dependently attenuated the secreted levels of TNF-α, IL-6, and IL-1β induced by LPS (Figure 2A). In line with the ELISA results, the qPCR and Western blot analysis shown that the expression levels of TNF-α, IL-6, and IL-1β were dramatically decreased by EFR pretreatment in a dose-dependent manner (Figure 2B,C). Based on these findings, EFR was able to suppress the inflammatory activation stimulated by LPS.

### 2.3. EFR Inhibits LPS-Induced Nuclear Translocation of NF-κB p65 and AP-1

**Figure 3:**
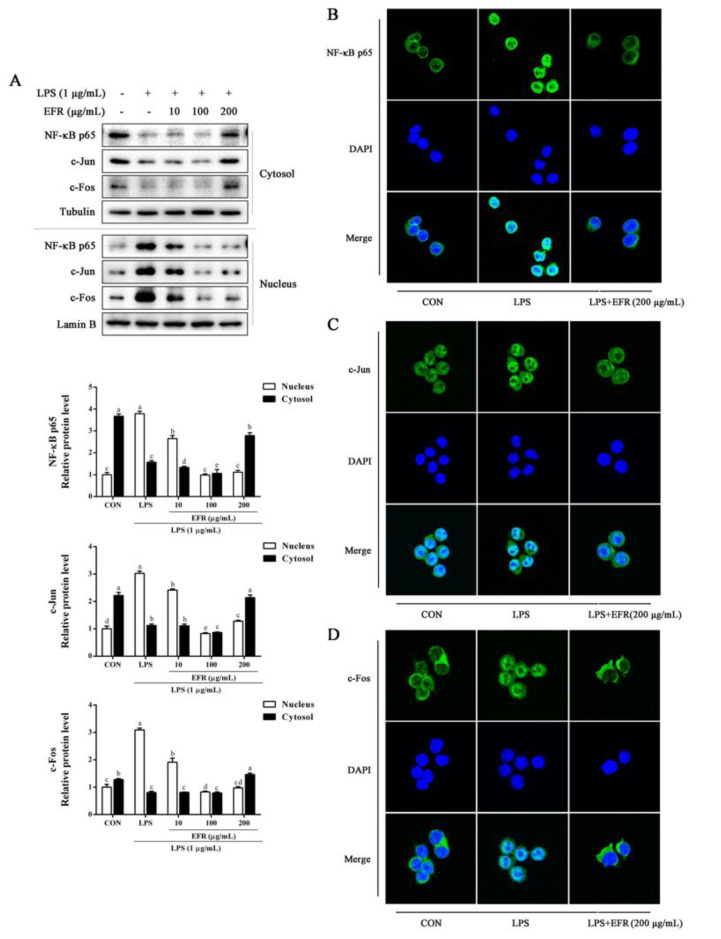
Effect of EFR on LPS-induced NF-κB p65 and AP-1 nuclear translocation in RAW 264.7 macrophages. (**A**) RAW 264.7 macrophages were pretreated with EFR (10, 100, and 200 μg/mL) for 2 h and then stimulated with or without LPS (1 μg/mL) for 30 min. The nuclear and cytosolic extracts of cells were prepared, and the protein levels of NF-κB p65, c-Jun, and c-Fos were determined by Western blot analysis. Lamin B and Tubulin were used as endogenous controls for the nucleus and cytoplasm, respectively. Relative intensity of the immunoreactive bands was analyzed using the Image J software. RAW 264.7 macrophages were pretreated with EFR (200 μg/mL) for 2 h and then stimulated with or without LPS (1 μg/mL) for 30 min. The cellular localization of NF-κB p65 (**B**), c-Jun (**C**), and c-Fos (**D**) was determined by immunofluorescence assay. Results are shown as the mean ± SD of three independent experiments. The different letters represent the statistical differences at *p* < 0.05 among the groups by Duncan’s multiple range test.

NF-κB and AP-1, two pivotal transcription factors that regulate the production of inflammatory mediators and cytokines, play essential roles in the inflammatory responses [24,25]. Herein, we investigated the effect of EFR on the nuclear translocation of NF-κB p65 and AP-1 in LPS-stimulated RAW 264.7 macrophages. As shown by Western blot analysis presented in Figure 3A, EFR pretreatment obviously decreased the translocation of NF-κB p65, c-Jun, and c-Fos from cytosol to nucleus triggered by LPS in a dose-dependent manner. Similarly, immunofluorescence assay also elucidated that LPS-induced nuclear translocation of NF-κB p65, c-Jun, and c-Fos was noticeably reversed by EFR pretreatment (Figure 3B–D). These results revealed that the inhibitory effect of EFR on the inflammatory responses was due to the inactivation of NF-κB and AP-1.

### 2.4. EFR Inhibits LPS-Induced Activation of NF-κB/MAPK Signaling Pathways

**Figure 4:**
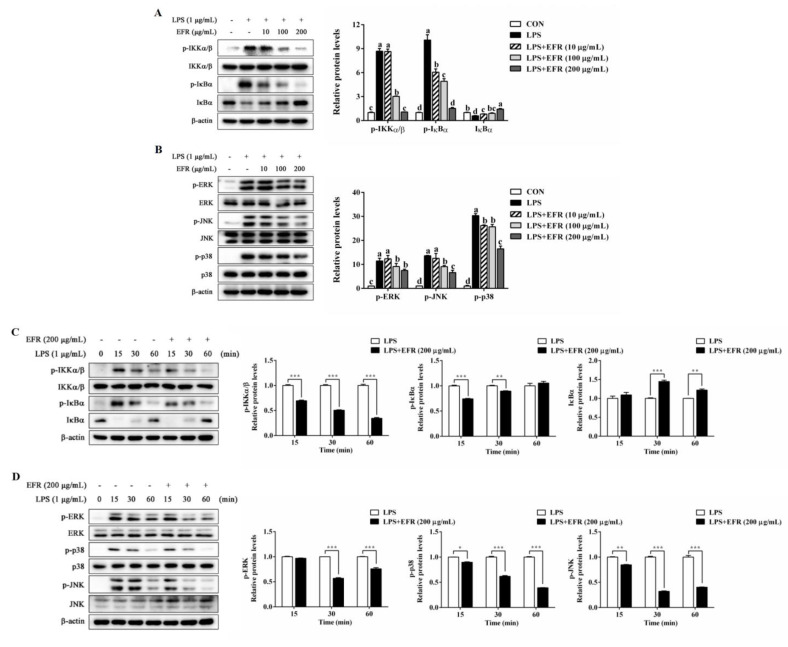
Effect of EFR on LPS-induced activation of NF-κB and MAPK signaling pathways in RAW 264.7 macrophages. RAW 264.7 macrophages were pretreated with EFR (10, 100, and 200 μg/mL) for 2 h and then stimulated with or without LPS (1 μg/mL) for 30 min. The protein levels of NF-κB (**A**) and MAPK (**B**) signaling pathways were determined by Western blot analysis. β-actin was used as an endogenous control. RAW 264.7 macrophages were pretreated with EFR (200 μg/mL) for 2 h, and then the total protein was harvested at different time points (15, 30, and 60 min) after stimulation with or without LPS (1 μg/mL), and the protein levels of NF-κB (**C**) and MAPK (**D**) signaling pathways were determined by Western blot analysis. β-actin was used as an endogenous control. Relative intensity of the immunoreactive bands was analyzed using the Image J software. Results are shown as the mean ± SD of three independent experiments. The different letters represent the statistical differences at *p* < 0.05 among the groups by Duncan’s multiple range test. * *p* < 0.05, ** *p* < 0.01, and *** *p* < 0.001, compared to the LPS-treated group.

To assess the molecular mechanism underlying the inactivation of NF-κB, we firstly investigated the effect of EFR on LPS-induced NF-κB and MAPK signaling pathways, which are known as two classical and critical inflammatory pathways. As illustrated in Figure 4A,C, EFR pretreatment significantly suppressed LPS-induced degradation of IκBα, as well as phosphorylation of IKKα/β and IκBα in a dose- and time-dependent manner, directly suggesting the inactivation of NF-κB. MAPKs have been reported to regulate the transcription factors of NF-κB and AP-1 in LPS-stimulated macrophages [11,12]. Our Western blot data also demonstrated that LPS-induced phosphorylation of ERK, JNK, and p38 was obviously attenuated by EFR pretreatment (Figure 4B,D). These results collectively suggested that NF-κB and MAPK signaling pathways were involved in EFR-mediated anti-inflammation.

### 2.5. EFR Activates AMPK by up-Regulating the ADP:ATP Ratio

**Figure 5:**
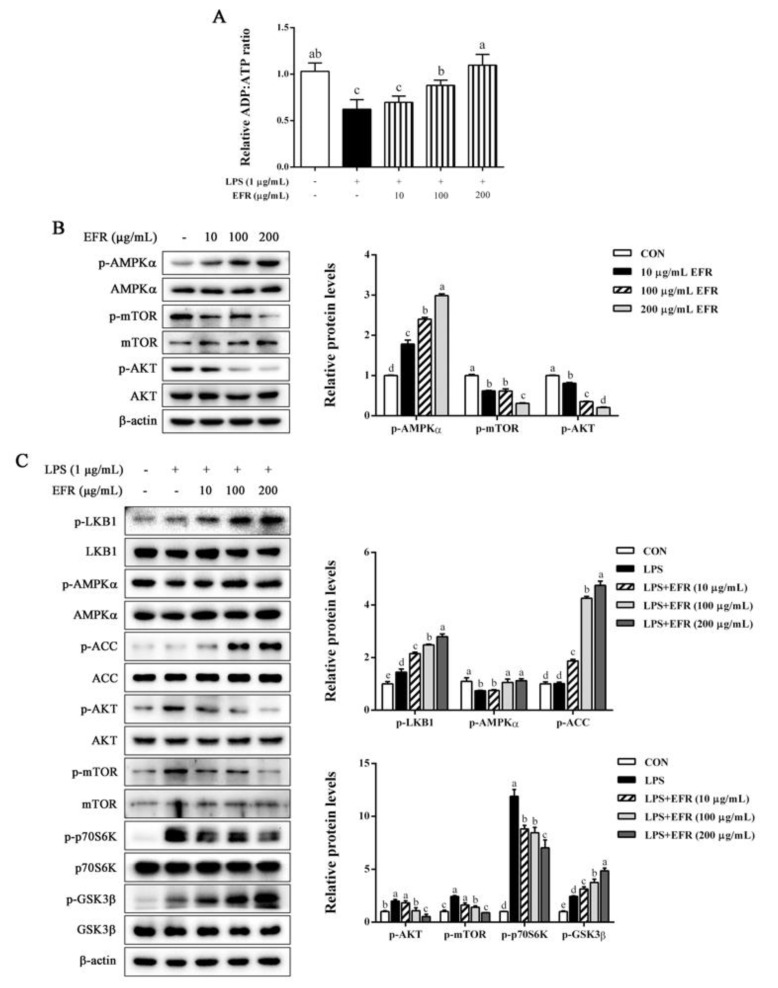
Effect of EFR on AMPK activation in LPS-stimulated RAW 264.7 macrophages. RAW 264.7 macrophages were pretreated with EFR (10, 100, and 200 μg/mL) for 2 h and then stimulated with or without LPS (1 μg/mL) for 30 min. (**A**) The ADP:ATP ratio was measured. The protein levels of p-AMPK*α*, AMPK*α*, p-mTOR, mTOR, p-AKT, AKT (**B**), and AMPK signaling pathways (**C**) were determined by Western blot analysis. *β*-actin was used as an endogenous control. Relative intensity of the immunoreactive bands was analyzed using the Image J software. Results are shown as the mean ± SD of three independent experiments. The different letters represent the statistical differences at *p* < 0.05 among the groups by Duncan’s multiple range test.

It is reported that the increased AMP/ADP:ATP ratio causes AMPK activation and in turn negatively regulates NF-κB activation [26,27]. To further clarify the specific upstream signaling pathway responsible for EFR-mediated suppression of NF-κB and MAPKs, we next investigated the effect of EFR on the ADP:ATP ratio and AMPK activation. As presented in Figure 5A, LPS stimulation resulted in the decreased ADP:ATP ratio as compared to the control, whereas EFR pretreatment significantly recovered the ADP:ATP ratio in a dose-dependent manner. In accordance with the changes in the ADP:ATP ratio, EFR treatment promoted the phosphorylation of AMPK*α*, indicating the activation of AMPK (Figure 5B). Furthermore, in agreement with previous studies, LPS treatment caused the inactivation of AMPK, as evidenced by the decreased phosphorylation of AMPK*α* and increased phosphorylated levels of mTOR, AKT, and p70S6K. However, all these alterations were prominently and dose-dependently blocked by EFR pretreatment (Figure 5C). Based on these results, it can be concluded that AMPK activation modulated by the change in ADP:ATP ratio may be related to the anti-inflammatory mechanism of EFR.

### 2.6. AMPK Activation Is Necessary for EFR-Mediated Suppression of NF-κB/MAPK Signaling Pathways

**Figure 6:**
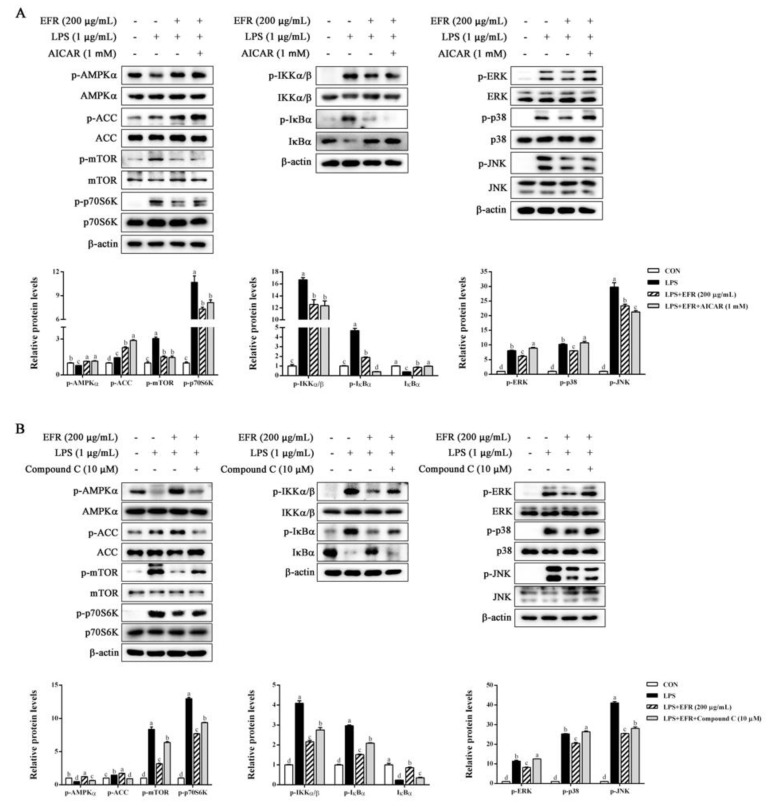
The role of AMPK activation in the EFR-mediated anti-inflammatory effect in LPS-stimulated RAW 264.7 macrophages. (**A**) RAW 264.7 macrophages were pretreated with or without AICAR (1 mM) for 2 h, then with or without EFR (200 μg/mL) for 2 h and stimulated with or without LPS (1 μg/mL) for 30 min. The protein levels of AMPK, NF-κB, and MAPK signaling pathways were determined by Western blot analysis. β-actin was used as an endogenous control. (**B**) RAW 264.7 macrophages were pretreated with or without Compound C (10 μM) for 2 h, then with or without EFR (200 μg/mL) for 2 h and stimulated with or without LPS (1 μg/mL) for 30 min. The protein levels of AMPK, NF-κB, and MAPK signaling pathways were determined by Western blot analysis. β-actin was used as an endogenous control. Relative intensity of the immunoreactive bands was analyzed using the Image J software. Results are shown as the mean ± SD of three independent experiments. The different letters represent the statistical differences at *p* < 0.05 among the groups by Duncan’s multiple range test.

To further ascertain the role of AMPK activation in the EFR-mediated anti-inflammatory mechanism, we first compared the inhibitory effect of EFR and that of the AMPK activator 5-aminoimidazole-4-carboxamide1-β-D-ribofuranoside (AICAR) on LPS-induced changes in AMPK, NF-κB, and MAPK signaling pathways. As shown in Figure 6A, both EFR and AICAR effectively recovered LPS-induced inactivation of the AMPK signaling pathway and activation of the NF-κB signaling pathway. However, in the MAPK signaling pathway, AICAR had no significant effects on the phosphorylation of ERK and p38. We next evaluated the effect of EFR on LPS-induced NF-κB and MAPK signaling pathways under the circumstance of AMPK inactivation using the AMPK-specific inhibitor Compound C. As depicted in Figure 6B, EFR-induced phosphorylation of AMPK*α* and ACC, and dephosphorylation of mTOR was abolished following the combined treatment with Compound C, implying the successive inactivation of AMPK. Meanwhile, as expected, EFR could not attenuate LPS-induced phosphorylation of IKKα/β and IκBα and subsequent degradation of IκBα in the present of Compound C. In parallel, the inhibitory effect of EFR on the activation of the MAPK pathway stimulated by LPS was prominently abrogated by AMPK inactivation. These data definitely authenticated that AMPK activation was indispensable for the anti-inflammatory effect of EFR in response to LPS treatment.

### 2.7. Identification of Anti-Inflammatory Compounds in EFR

**Figure 7:**
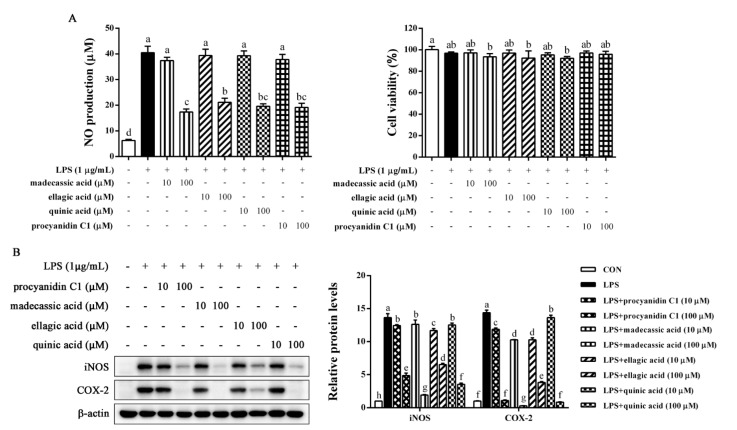
Inhibitory effect of major compounds in EFR on LPS-induced inflammatory responses in RAW 264.7 macrophages. RAW 264.7 macrophages were pretreated with madecassic acid, ellagic acid, quinic acid, and procyanidin C1 (10 and 100 μM) for 2 h and then stimulated with or without LPS (1 μg/mL) for 24 h. (**A**) The nitrite concentration in the cultured media was determined as an indicator of NO production by Griess reaction. (**B**) The protein levels of iNOS and COX-2 were determined by Western blot analysis. β-actin was used as an endogenous control. Relative intensity of the immunoreactive bands was analyzed using the Image J software. Results are shown as the mean ± SD of three independent experiments. The different letters represent the statistical differences at *p* < 0.05 among the groups by Duncan’s multiple range test.

Chemical composition of EFR was further analyzed by LC–MS (Figure S1, Supplementary Materials). The top 20 components with relative abundance are shown in Table 1. We preliminarily examined the anti-inflammatory property of components which are commercially available by the inhibitory effects on LPS-induced NO production and found that only madecassic acid, ellagic acid, quinic acid, and procyanidin C1 were obviously effective. Therefore, these four compounds were chosen for further verification. As can be seen in Figure 7A, madecassic acid, ellagic acid, quinic acid, and procyanidin C1 exerted a remarkable inhibitory effect on LPS-induced NO production, hardly affecting the viability of tested cells. Furthermore, similar results were observed in protein levels of iNOS and COX-2 (Figure 7B). Our finding indicated that the presence of these four compounds in EFR may be a part of contributing factors responsible for EFR’s anti-inflammatory activities.

## 3. Discussion

Natural products or bioactive compounds from plant materials, especially traditional Chinese herbs, have attracted widespread attention in terms of the development of beneficial dietary supplements and therapeutic drug candidates owing to their medicinal properties with low toxicity and side effects. Previous studies have reported that the total flavonoids or saponins from *Rosa laevigata Michx*. fruit exert anti-inflammatory effect by inhibiting NF-κB transcriptional activities [19,20,21,28,29]. However, how NF-κB is affected, or in other words, the exact anti-inflammatory mechanism remains unclear. In this study, we demonstrated for the first time that EFR effectively restrained inflammatory responses in LPS-stimulated RAW 264.7 macrophages through NF-κB/MAPK signaling pathways via AMPK activation. Additionally, madecassic acid, ellagic acid, quinic acid, and procyanidin C1 were proved to be some of the active components responsible for EFR’s anti-inflammatory actions. These results indicated an accurate anti-inflammatory mechanism and the potential of EFR to be a therapeutic drug candidate for inflammatory diseases.

Redundant inflammatory mediators (NO and PGE_2_) and cytokines (TNF-α, IL-6, and IL-1β) produced by activated macrophages have been demonstrated to aggravate the progress of inflammatory damage and ultimately result in auto-inflammatory and autoimmune disorders [30,31]. Therefore, evaluating the inhibitory effect of natural products or compounds on the production of these inflammatory markers could be a feasible method for developing alternative immunosuppressive and anti-inflammatory therapeutic candidates. In the present work, we found that EFR-mediated suppression of the excessive production of NO and PGE_2_ in LPS-stimulated RAW 264.7 macrophages was attributed to the down-regulated expression of iNOS and COX-2 at both protein and mRNA levels (Figure 1). In addition, EFR was also shown to effectively inhibit LPS-induced TNF-α, IL-6, and IL-1β production (Figure 2). Based on these results, we suggested that EFR may effectively attenuate inflammatory responses through the suppression of inflammatory mediators and cytokines production in LPS-stimulated RAW 264.7 macrophages.

NF-κB is considered to be the most decisive nuclear transcription factor that mediates the production of inflammatory mediators and cytokines during inflammatory responses [24,32]. Numerous studies have illustrated that many anti-inflammatory agents exhibit their potency by the suppression of the NF-κB signaling pathway [4,22,23]. The activation of NF-κB occurs by the IKK-mediated phosphorylation and proteolytic degradation of IκBα. Then, activated cytosolic NF-κB dimer (p65/p50) translocates into the nucleus and activates the transcription of the targeted genes such as COX-2 and iNOS [9,10,33]. In this study, our Western blot analysis revealed that the phosphorylation of IKKα/β and IκBα, as well as the degradation of IκBα induced by LPS was effectively reversed by EFR pretreatment (Figure 4). Further Western blot and immunofluorescence analysis indicated that preincubation with EFR significantly recovered LPS-induced nuclear translocation of NF-κB p65 subunit, supporting the inactivation of NF-κB by EFR (Figure 3A,B). Similar results were also obtained from the analysis of nuclear translocation of AP-1 (Figure 3C,D). Evidence has shown that the MAPK pathway, among the most ancient and highly conserved signaling pathways, has been proved to implement vital roles in inflammatory and immune responses, and the activation of JNK, ERK, and p38 by phosphorylation is closely associated with the expression of iNOS, COX-2, and cytokine genes by promoting NF-κB signaling events [34,35]. According to our results, the phosphorylation of JNK, ERK, and p38 induced by LPS was expressively alleviated by EFR pretreatment (Figure 4). Herein, it is likely that the suppression of MAPKs phosphorylation may contribute to EFR-mediated inhibition of the NF-κB signaling pathway.

Despite the ongoing research, the specific target by which EFR regulated the NF-κB/MAPK signaling pathways was not fully revealed. Therefore, we next explored the possible upstream regulating signaling molecules and pathways. AMPK is the major cellular energy sensor and regulator of metabolic homeostasis, consisting of a catalytic α subunit and regulator β and γ subunits [36,37]. AMPK is activated by increased AMP/ADP:ATP ratio and phosphorylation of the α subunit at Thr172 by upstream kinases such as LKB1 [38,39]. Previous studies have indicated that AMPK activation is highly associated with the regulation of inflammatory responses via the suppression of NF-κB and MAPK signaling pathways [15,40,41]. Thus, we turned our attention to examine whether AMPK activation was involved in the EFR-mediated anti-inflammatory mechanism. The present study showed that EFR could activate AMPK possibly through the elevated ADP:ATP ratio, resulting in the suppression of LPS-induced activation of NF-κB and MAPK signaling pathways (Figure 5). We also found that AICAR, an agonist of AMPK, exerted its effectiveness similar to that of EFR (Figure 6A). Additionally, EFR could not alleviate LPS-induced inflammatory responses in the presence of Compound C (Figure 6B). These results suggested that EFR exerted anti-inflammatory effect via AMPK activation.

Previous phytochemical and pharmacological studies indicated the presence of polysaccharides, triterpenoids, flavonoids, and saponins in *Rosa laevigata Michx*. fruit, which was shown to have hepatoprotective, neuroprotective, anti-oxidant and anti-inflammatory activities [21,28,42,43]. However, we found via literature searches that there was few research systematically revealing the anti-inflammatory mechanism and chemical composition responsible for the anti-inflammatory actions. In this study, LC–MS was conducted to analyze the chemical composition of EFR (Figure S1). Madecassic acid, ellagic acid, quinic acid, and procyanidin C1 were identified and proved to show a remarkable inhibitory effect on LPS-stimulated NO production and inflammatory mediator expression (Figure 7A,B). Many studies have indicated the anti-inflammatory effects of these four compounds. Madecassic acid was reported to inhibit LPS-induced iNOS, COX-2, TNF-α, IL-1β, and IL-6 expression via the down-regulation of NF-κB activation in RAW 264.7 macrophage cells [44]. Ellagic acid has been demonstrated to suppress the inflammatory responses in keratinocytes by regulating MAPK and STAT signaling pathways [45]. It was shown that quinic acid could inhibit vascular inflammation in TNF-α-stimulated vascular smooth muscle cells by suppressing the MAP kinase and NF-κB signaling pathways [46]. Procyanidin C1 was clarified to exert an anti-inflammatory effect by regulating LPS-induced MAPK and NF-κB signaling through TLR4 in macrophages [47]. Based on experimental verification combined with literature searches, the presence of these four compounds, as well as other unidentified components, may contribute to EFR’s anti-inflammatory effects.

## 4. Materials and Methods

### 4.1. Plant Material and Extraction Preparation

The *Rosa laevigata Michx*. fruit was purchased from Anhui Zhixin Zhongyao Yinpian Co., Ltd. (Anhui, China). The extraction process was conducted via cold maceration with the sample to solvent ratio of 1:10. Briefly, the dried powder (100 g) was extracted with 95% ethanol (1 L) for 12 h at 4 °C, the decoction was filtered, concentrated using a rotary vacuum evaporator (N-1200BV, EYELA, Tokyo, Japan) at 55 °C, and then lyophilized by a freeze-dryer (FDU-1200, EYELA, Tokyo, Japan). The extract was kept at −20 °C for later experiments.

### 4.2. Chemicals and Reagents

Lipopolysaccharide (LPS), 3-(4,5-dimethylthiazol-2yl)-2,5-diphenyltetrazolium bromide (MTT), AICAR, and Compound C were purchased from Sigma-Aldrich (St. Louis, MO, USA). Oligonucleotides were synthesized by Sangon Biotech (Shanghai, China). TRIzol, Dulbecco’s Modified Eagle’s Medium (DMED), fetal bovine serum (FBS), penicillin, and streptomycin were purchased from Invitrogen (Carlsbad, CA, USA). All other chemicals and reagents were analytical purity and commercially available.

### 4.3. Cell Culture

The murine macrophage cell line RAW 264.7 was purchased from the Shanghai Cell Bank of the Chinese Academy of Sciences (Shanghai, China). Cells were cultured in DMEM with 10% FBS, 100 U/mL penicillin, and 100 μg/mL streptomycin in a humidified atmosphere with 5% CO_2_ at 37 °C.

### 4.4. MTT Assay

Cell viability was determined using the MTT assay. RAW 264.7 macrophages (2 × 10^4^ cells/well) were seeded in 96-well plates to adhere overnight. Cells were pretreated with EFR (10, 100, and 200 μg/mL) for 2 h and then stimulated with or without LPS (1 μg/mL) for 24 h. Then, 10 μL of 5 mg/mL MTT were added in each well, followed by another 4 h incubation. The cultured media were discarded, and 150 μL DMSO were added to dissolve the formazan crystals. Absorbance of formazan solution was measured at 490 nm using a SpectraMax^®^ i3x Multi-Mode Microplate Reader (Molecular Devices, CA, USA).

### 4.5. Griess Assay

RAW 264.7 macrophages (2 × 10^4^ cells/well) were seeded in 96-well plates to adhere overnight. Cell were pretreated with EFR (10, 100, and 200 μg/mL) for 2 h and then stimulated with or without LPS (1 μg/mL) for 24 h. The cultured media were collected and centrifugated. The nitrite concentration in the cultured supernatants was measured as an indicator of NO production using the Nitric Oxide Assay Kit (Beyotime Institute of Biotechnology, Shanghai, China) according to the manufacturer’s instructions.

### 4.6. Enzyme-Linked Immunosorbent Assay (ELISA)

RAW 264.7 macrophages (4 × 10^5^ cells/well) were seeded in 12-well plates to adhere overnight. Cells were pretreated with EFR (10, 100, and 200 μg/mL) for 2 h and then stimulated with or without LPS (1 μg/mL) for 24 h. The cultured media were collected and centrifugated. The level of PGE_2_ in the cultured supernatants was quantitated by the Prostaglandin E_2_ Parameter Assay Kit (R&D Systems, Minneapolis, MN, USA), and levels of TNF-α, IL-6, and IL-1β were determined using the ELISA kits (ABclonal Biotechnology, Wuhan, China) according to the manufacturer’s instructions.

### 4.7. Measurement of Cellular ADP: ATP Ratio

RAW 264.7 macrophages (4 × 10^5^ cells/well) were seeded in 12-well plates to adhere overnight. Cells were pretreated with EFR (10, 100, and 200 μg/mL) for 2 h and then stimulated with or without LPS (1 μg/mL) for 30 min. The cellular ADP:ATP ratio was measured using the ADP:ATP Ratio Assay Kit (Abcam, Cambridge, UK) according to the manufacturer’s instructions.

### 4.8. Preparation of Nuclear and Cytosolic Extracts

RAW 264.7 macrophages (5 × 10^6^ cells/well) were seeded in each 60 mm dish to adhere overnight. Cells were pretreated with EFR (10, 100, and 200 μg/mL) for 2 h and then stimulated with or without LPS (1 μg/mL) for 30 min. The nuclear and cytosolic extracts of the cells were prepared using the Nuclear and Cytoplasmic Protein Extraction Kit (Beyotime Institute of Biotechnology, Shanghai, China) according to the manufacturer’s instructions.

### 4.9. Total RNA Extraction, Reverse Transcription, and Quantitative Real-Time PCR Analysis

Total RNAs of RAW 264.7 macrophages were isolated using TRIzol reagent. Then, 1 μg of total RNA was reverse transcribed to cDNA using the HiFi-MMLV cDNA kit (CoWin Biosciences, Beijing, China) according to the manufacturer’s instructions. Quantitative Real-time PCR analysis was performed by the Roche Light Cycler^®^ 480 System (Roche Group, Switzerland) using the Ultra SYBR Mixture (CoWin Biosciences, Beijing, China) referring to the protocol. The following sequences of specific primers were used in the present study. iNOS: Forward 5′-CAACCAGTATTATGGCTCCT-3′; Reverse 5′-GTGACAGCCCGGTCTTTCCA-3′, COX-2: Forward 5′-CAGCAAATCCTTGCTGTTCC-3′; Reverse 5′-TGGGCAAAGAATGCAAACATC-3′, TNF-α: Forward 5′-AGCCGATGGGTTGTACCTTG-3′; Reverse 5′-ATAGCAAATCGGCTGACGGT-3′, IL-6: Forward 5′-GAGTGGCTAAGGACCAAGACC-3′; Reverse 5′-AACGCACTAGGTTTGCCGA-3′, IL-1β: Forward 5′-TCCAGGATGAGGACATGAGCAC-3′; Reverse 5′-GAACGTCACACACCAGCAGGTTA-3′, GAPDH: Forward 5′-AAACGGCTACCACATCCAAG-3′; Reverse 5′-CCTCCAATGGATCCTCGTTA-3′.

Thermocycler conditions included an initial denaturation at 94 °C for 3 min, 40 cycles of denaturation at 94 °C for 30 s, annealing at 60 °C for 30 s, and extension at 72 °C for 30 s, followed by a 2 min extension at 72 °C. The results were analyzed using the 2^−ΔΔCt^ method. The relative gene expression was normalized to GAPDH expression.

### 4.10. Immunofluorescence Assay

RAW 264.7 macrophages (4 × 10^5^ cells/well) were seeded on coverslips in 12-well plates to adhere overnight. Cells were pretreated with EFR (200 μg/mL) for 2 h and then stimulated with or without LPS (1 μg/mL) for 30 min. After that, cells were fixed with 4% paraformaldehyde for 20 min, treated with 0.2% Triton-X-100 for 10 min, and then blocked with 5% bovine serum albumin (BSA) in PBS for 1 h. Thereafter, cells were incubated with primary antibodies for NF-κB p65 (1:400), c-Jun (1:100), and c-Fos (1:200) at 4 °C overnight, and then washed with PBS twice before incubation with donkey anti-rabbit IgG, Alexa Fluor 488 (1:500) (Thermo Fisher Scientific, Waltham, MA, USA) for 1 h at room temperature. After washing twice with PBS, the nuclei were stained with DAPI Staining Solution (Beyotime Institute of Biotechnology, Shanghai, China) in the dark. Images were captured using a Leica TCS SP8 Confocal Microscope (Leica Microsystems GmbH, Wetzlar, Germany).

### 4.11. Western Blot Analysis

Total proteins of RAW 264.7 macrophages were extracted and the protein concentrations were determined using the Enhance BCA Protein Assay Kit (Beyotime Institute of Biotechnology, Shanghai, China) according to the manufacturer’s instructions. Then, aliquots of proteins (20~40 μg) were separated by sodium dodecyl sulfate-polyacrylamide gel electrophoresis (SDS-PAGE) and transferred onto polyvinylidene diflfluoride (PVDF) membranes (Millipore Corp., Billerica, MA, USA). The membranes were blocked with 5% BSA in Tris-buffered saline with Tween-20 (TBST) for 1 h at room temperature and then incubated with a 1:1000 dilution of various primary antibodies in 5% BSA with TBST at 4 °C overnight. Antibodies against IL-6, IKKα/β, p-IKKα/β, IκBα, p-IκBα, JNK, p-JNK, ERK, p-ERK, p38, p-p38, AMPKα, p-AMPKα, ACC, p-ACC, AKT, p-AKT, mTOR, p-mTOR, p70S6K, and p-p70S6K were purchased from Cell Signaling Technology (Beverly, MA, USA). Antibodies against iNOS, COX-2, NF-κB p65, LKB1, p-LKB1, GSK3β, p-GSK3β c-Jun, c-Fos, α-Tubulin, Lamin B, and β-actin were purchased from ABclonal Biotechnology (Wuhan, China). Antibodies against TNF-α and IL-1β were purchased from Proteintech Group (Wuhan, China). The membranes were washed three times with TBST and then incubated with horseradish peroxidase-conjugated secondary antibodies to rabbit IgG or to mouse IgG (1:5000) purchased from Jackson ImmunoResearch Laboratories (West Grove, PA, USA) for 1 h at room temperature. After washing three times with TBST, the immunoreactive proteins were visualized using the NcmECL Ultra (NCM Biotech, Suzhou, China) according to the manufacturer’s instructions and then imaged using the ChemiDoc™ XRS+ System (Bio-Rad, Hercules, CA, USA).

### 4.12. LC–MS Detection

The Waters Acquity I-Class PLUS ultra-high-performance liquid tandem Waters Xevo G2-XS QTOF high-resolution mass spectrometer (Waters Corporation, Milford, MA, USA) was used for metabolite analysis of EFR. UPLC fitted the Waters Acquity UPLC HSS T3 column (1.8 μm, 2.1 × 100 mm). The parameter was performed as follows: mobile phase A, 0.1% formic acid in water; mobile phase B, 0.1% formic acid in acetonitrile; gradient with 2% mobile phase B for 0~0.25 min, 2~98% mobile phase B for 0.25~10 min, 98% mobile phase B for 10~13 min, 98~2% mobile phase B for 13~13.1 min, 2% mobile phase B for 13.1~15 min. The parameters of the electrospray ionization-mass spectrometry (ESI-MS) analysis were 2.0 kV (positive ion mode) and −1.5 kV (negative ion mode), cone voltage of 30 V, ion source temperature of 150 °C, desolvent gas temperature of 500 °C, backflush gas flow rate of 50 L/h, and desolventizing gas flow rate of 800 L/h. MassLynx V4.2 with MSe mode (Waters) was utilized for collection of primary and secondary mass spectrometry data. The peak extraction, alignment and data processing operations were conducted by Progenesis QI software. The METLIN database and Biomark’s self-built library (Biomarker Technologies Co., Ltd., Beijing, China) were used for metabolite identification.

### 4.13. Statistical Analysis

Results are expressed as the mean ± standard deviation (SD) and all assays were performed at least in triplicate. Duncan’s multiple range test of one-way analysis of variance (ANOVA) was performed by IBM SPSS Statistics 20 software package (SPSS Inc. Chicago, IL, USA) at a confidence level of 95%. Other statistical evaluations were performed by Student’s *t*-test. A *p* value < 0.05 was considered statistically significant.

## 5. Conclusions

In summary, the findings of this study enriched the anti-inflammatory mechanism of EFR, which has been demonstrated to inhibit inflammatory responses through NF-κB/MAPK signaling pathways via AMPK activation, and represents an effective therapeutic intervention for the treatment of inflammatory disease.

## Figures and Tables

**Table 1:** Identification of the top 20 components with relative abundance in EFR by LC–MS (*n* = 3).

Compounds	Formula	*m*/*z*	Retention Time (min)	Ion Mode
Myrianthic acid	C_30_H_48_O_6_	469.3293	6.4002	pos
Glabrolide	C_30_H_44_O_4_	469.3313	4.7757	pos
Madecassic acid	C_30_H_48_O_6_	503.3342	5.9403	neg
16*b*-16-Hydroxy-3-oxo-1,12-oleanadien-28-oic acid	C_30_H_44_O_4_	469.3311	4.1621	pos
Ganolucidic acid B	C_30_H_46_O_6_	503.3361	6.2071	pos
N6-(l-1,3-Dicarboxypropyl)-l-lysine	C_11_H_20_N_2_O_6_	276.1455	1.5622	pos
PC(18:1(9E)/0:0)[U]	C_26_H_52_NO_7_P	522.3557	8.3217	pos
1,2-Dimethoxy-13-methyl-[1,3]benzodioxolo [5,6-*c*]phenanthridine	C_21_H_17_NO_4_	348.1243	4.6405	pos
Ellagic acid	C_14_H_6_O_8_	301.0002	3.4479	neg
Ile Ser Arg Lys	C_21_H_42_N_8_O_6_	501.3214	6.0690	neg
Tomentosolic acid	C_30_H_46_O_3_	437.3411	8.2216	pos
PC(16:0/0:0)[U]/PC(16:0/0:0)[rac]	C_24_H_50_NO_7_P	496.3406	8.0506	pos
4,4′-Methylenebis (2,6-di-tert-butylphenol)	C_29_H_44_O_2_	425.3429	5.0260	pos
Cyanidin 3-*O*-rutinoside	C_27_H_31_O_15_	1189.2998	2.2124	neg
Quinic acid	C_7_H_12_O_6_	191.0577	0.7015	neg
Procyanidin C1	C_45_H_38_O_18_	865.1970	2.6351	neg
5-Acetylamino-6-formylamino-3-methyluracil	C_8_H_10_N_4_O_4_	451.1260	2.4086	neg
(−)-Catechin	C_15_H_14_O_6_	289.0696	2.7075	neg
1-Oleoyl-sn-glycero-3-phosphocholine	C_26_H_52_NO_7_P	566.3457	8.3592	neg
Neriifolin	C_30_H_46_O_8_	533.3122	5.2195	neg

## Data Availability

The data used to support the findings of this work are available from the corresponding author upon request.

## Supplementary Materials

The following supporting information can be downloaded at: https://www.mdpi.com/article/10.3390/molecules28062813/s1, Figure S1: LC–MS analysis of the chemical composition of EFR in positive and negative modes (*n* = 3).

## Sample Availability

Samples of the EFR are available from the authors.
