# Supplementary figures and images for: Ethanol Extract of Rosa laevigata Michx. Fruit Inhibits Inflammatory Responses through NF-κB/MAPK Signaling Pathways via AMPK Activation in RAW 264.7 Macrophages
Source: Molecules. 2023 Mar 20;28(6):2813. doi: 10.3390/molecules28062813 (PMC10054580; doi:10.3390/molecules28062813)

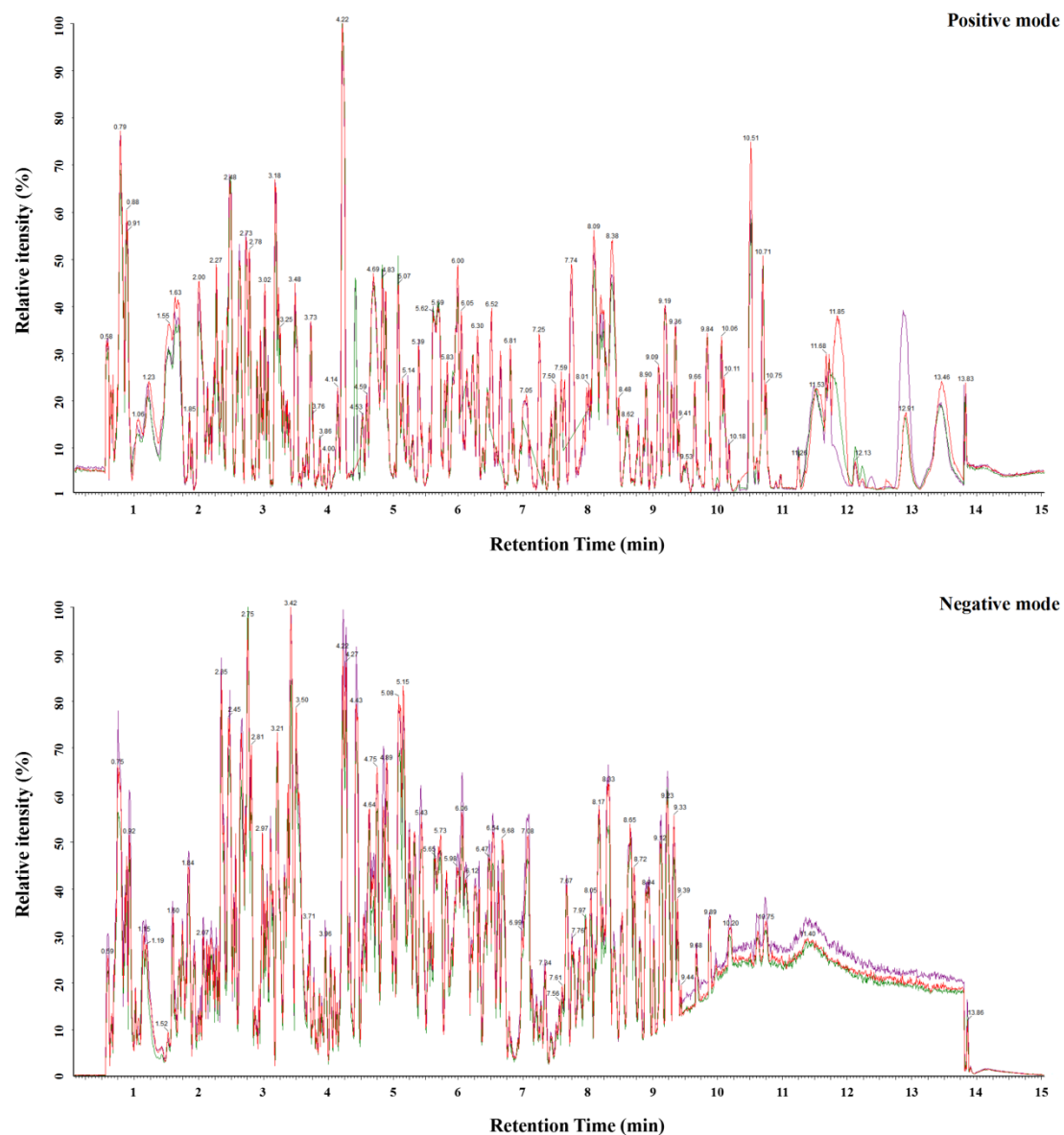

**Figure S1.** LC/MS analysis of the chemical composition of EFR in positive and negative modes ( $n=3$ ).

Supplement: Supplementary file 1 [file molecules-28-02813-s001.zip › molecules-2292187-supplementary.pdf]
